# Estimation of the incidence of severe fever with thrombocytopenia syndrome in high endemic areas in China: an inpatient-based retrospective study

**DOI:** 10.1186/s12879-018-2970-7

**Published:** 2018-02-05

**Authors:** Xiaoxia Huang, Shiwen Wang, Xianjun Wang, Yong Lyu, Mei Jiang, Deying Chen, Kaichun Li, Jingyu Liu, Shaoyu Xie, Tao Lyu, Jie Sun, Pengpeng Xu, Minghua Cao, Mifang Liang, Dexin Li

**Affiliations:** 1 National Institute for Viral Disease Control and Prevention, Chinese Center for Disease Control and Prevention, 155 Changbai Road, Changping District, Beijing, 102206 People’s Republic of China; 2 Shandong Center for Disease Control and Prevention, Jinan, Shandong Province People’s Republic of China; 3 Lu’an Center for Disease Control and Prevention, Lu’an, Anhui Province People’s Republic of China; 4 Yantai Center for Disease Control and Prevention, Yantai, Shandong Province People’s Republic of China; 5 Weihai Center for Disease Control and Prevention, Weihai, Shandong Province People’s Republic of China; 6 Anhui Center for Disease Control and Prevention, Hefei, Anhui Province People’s Republic of China

**Keywords:** SFTS, SFTSV antibodies, Missed diagnosis, SFTS incidence

## Abstract

**Background:**

Severe fever with thrombocytopenia syndrome (SFTS) is a severe viral disease caused by SFTSV. It is important to estimate the rate of missed SFTS diagnosis and to further understand the actual incidence in high endemic areas in China.

**Methods:**

This study was conducted in two high SFTS endemic provinces in 2015. Patients hospitalized in 2014 or within 1 year before investigation were selected after considering their clinical manifestations, specifically, fever, platelet, and white blood cell. During retrospective investigation, sera were collected to detect SFTSV antibodies to assess SFTSV infection. To further understand SFTSV infection, acute phase sera were detected; SFTSV infection rate among a healthy population was also investigated to determine the basic infection level.

**Results:**

In total, 246 hospitalized cases were included, including 83 cases (33.7%) with fever, thrombocytopenia and leukopenia, 38 cases (15.4%) with fever and thrombocytopenia but without leukopenia, and 125 cases (50.8%) without fever but with thrombocytopenia and leukopenia. In total, 13 patients (5.3%) were SFTSV IgM antibody-positive, 48 (19.5%) were IgG-positive. Of the 13 IgM-positive cases, 11 (84.6%) were IgG-positive (9 with titres ≥1:400). Seropositive rates of antibodies were high (8.4% for IgM and 30.1% for IgG) in patients with fever, thrombocytopenia and leukopenia. Furthermore, among IgG-positive cases in this group, 76% (19/25) of patients’ IgG antibody titres were ≥1:400. Additionally, 28 of 246 cases were initially diagnosed with suspected SFTS and were then excluded, and 218 patients were never diagnosed with SFTS; the seropositive rates of IgM and IgG in these two groups were 25% and 67.9% and 2.8% and 13.3%, respectively. These rates were 64.3% and 21.4% in 14 sera collected during acute phase of the 28 cases mentioned above. Seropositive rate of SFTSV IgG was only 1.3% in the patient-matched healthy group, and no IgM antibody was detected. A preliminary estimate of 8.3% of SFTS cases were missed in SFTS high endemic provinces.

**Conclusions:**

The actual SFTS incidence was underestimated. Effective measures such as adding a new SFTS case category - “SFTS clinical diagnosis cases” or using serological detection methods during acute phase should be considered to avoid missed diagnoses.

**Electronic supplementary material:**

The online version of this article (10.1186/s12879-018-2970-7) contains supplementary material, which is available to authorized users.

## Background

Severe fever with thrombocytopenia syndrome (SFTS) is a newly discovered infectious disease that was reported in 2009 in two provinces of central China and had a 7.9% fatality rate [1, 2]. Thereafter, the SFTS case number increased annually [2]. With the expansion in endemic areas, 25 provinces reported having more than 10,000 SFTS cases by 2016. SFTS virus (SFTSV), a novel member of the *Phlebovirus* genus of the *Phenuiviridae* family, was identified to be its pathogen [1]. SFTSV can be transmitted by tick bites [3, 4]. Person-to-person transmission through blood or body fluid contact was also an important transmission route and caused multiple cluster cases in several provinces [5–12]. Because of the high mortality and person-to-person transmission, SFTS has become a serious threat to public health.

In China, more than 85% of SFTS cases are farmers living in seven provinces (Henan, Shandong, Hubei, Anhui, Liaoning, Zhejiang, and Jiangsu) [2]. The clinical manifestations of SFTS vary from mild symptoms to death [2, 4, 13, 14], although they mainly include fever, thrombocytopenia, leukopenia, and gastrointestinal symptoms; disseminated intravascular coagulation and multi-organ failure can occur in some severe cases [1, 4, 15–17]. A small portion of SFTS cases have a definitive history of a tick bite [18] and lymphadenopathy [1]. SFTS needs to be identified with other infectious diseases (haemorrhagic fever with renal syndrome, human anaplasmosis, leptospirosis, and septicaemia,) and non-infectious diseases (thrombocytolytic purpura) [1, 15].

In China, SFTS cases are officially divided into two categories: suspected cases and lab-confirmed cases [15]. In 2010, SFTS surveillance was conducted to understand its epidemic characteristics, and this surveillance was very important for providing basic epidemic data for SFTS prevention and control. However, accurate monitoring data depend on accurate disease diagnoses. Previous studies have shown that missed diagnoses [19–21] exist in many infectious diseases such as hepatitis B, measles, and rubella. However, the occurrence of missed diagnosis in SFTS was unclear. Therefore, we conducted this study to obtain rudimentary knowledge of SFTS missed diagnosis and to further understand the actual incidence of SFTS by studying the epidemiological features, clinical manifestations, and presence of antibodies against SFTSV in hospitalized patients.

## Methods

### Study sites

This study was conducted in two high SFTS endemic provinces. For confidentiality, we used “place1” and “place2” to represent these two provinces. In total, eight hospitals in these areas were selected to be included in this study. Moreover, an area that had no reported cases of SFTS before this investigation was selected as a control site to determine the SFTSV prevalence in a healthy population.

### SFTS case definition

According to the “Guideline for SFTS prevention and control (2010)” [15] issued by the National Health and Family Planning Commission of the People’s Republic of China, SFTS cases were divided into two categories: suspected cases and lab-confirmed cases. The diagnostic criteria for suspected cases included the following: an epidemiologic history (i.e., working, living or traveling in the hills, forest or mountain in SFTS epidemic season or being bitten by ticks within 2 weeks before disease onset) and clinical manifestations, such as fever and decrease in platelets and white blood cells in peripheral blood. Lab-confirmed SFTS cases were defined as suspected cases with at least one positive laboratory test (SFTSV RNA detected, seroconversion or four-fold increase in SFTSV IgG antibody or SFTSV isolated from specimens).

### Study subjects

In this study, subjects were required to be hospitalized patients in 2014 or within 1 year before the investigation and to have clinical characteristics in the following groups:

1. Fever (> 37.3 °C) with thrombocytopenia (< 100*10^9^/L) and leukopenia (< 4*10^9^/L);
2. Fever (> 37.3 °C) with thrombocytopenia (< 100*10^9^/L) and without leukopenia (< 4*10^9^/L); or
3. Thrombocytopenia (< 100*10^9^/L) and leukopenia (< 4*10^9^/L) without fever (> 37.3 °C).

If patients with the above characteristics were definitively diagnosed with immune or blood diseases (such as septicaemia and thrombocytolytic purpura) or were lab-confirmed SFTS cases, they were excluded from this study. Furthermore, the subjects were also required to be healthy during the retrospective investigation.

### Epidemiologic and clinical data collection

A retrospective survey was conducted with a constructed questionnaire designed to collect demographic information and exposure history. Clinical characteristics including time of disease onset, clinical diagnosis, body temperature, platelet (PLT) and white blood cell (WBC) counts, alanine aminotransferase (ALT), aspartate transaminase (AST), lactate dehydrogenase (LDH), and creatine kinase (CK) were retrieved from patients’ medical records.

### Sample collection and detection

During the investigation, blood samples were collected from the study subjects (including children participants) and sera were separated. Additionally, sera samples that had been collected from the subjects during hospitalization were analysed using SFTSV antibody and RNA tests. Enzyme-linked immunosorbent assay kits (Zhongshan bio-tech CO., LTD.) were used to detect antibodies (IgM and IgG) against SFTSV and to quantify IgG titres as described in the instructions. According to the detection instructions, 100 μl diluted sera (10 μl sera in 90 μl sample dilution for IgM detection, 1 μl sera diluted in 100 μl sample dilution for IgG detection) were added to each well and were incubated at 37 °C for 30-40 min. After washing, 100 μl horseradish peroxidase (HRP) - labelled reagent was placed in each well and incubated at 37 °C for 30-40 min. Then, chromogenic agents were added to develop colour change. After incubation, the optical density (OD) value was read at 450 nm. For every detection or plate, two negative and two positive controls were set. Based on an equation described previously [22], the cutoff value was calculated. A SFTSV IgM- or IgG-positive sample was defined as a sample with an OD value greater than or equal to the cutoff value. SFTSV RNA was detected by real-time reverse transcription-polymerase chain reaction (RT-PCR) method, as previously described [1].

### Statistical analysis

Statistical analysis was performed by SPSS 18.0 (Chicago, IL, USA), and the statistical significance was set at *p* < 0.05. Chi-square test (Pearson Chi-square or Fisher’s exact test) was used to analyse count data to compare the clinical manifestations and SFTSV antibody-positive rate in different groups.

## Results

### Hospitalized patients’ information

A total of 246 hospitalized patients were included in this study, including 83 cases (33.7%) with fever, thrombocytopenia and leukopenia, 38 cases (15.4%) with fever and thrombocytopenia but without leukopenia, and 125 patients (50.8%) without fever but with thrombocytopenia and leukopenia. Of the 246 hospitalized cases, 130 (52.8%) were male; the largest proportion, 180 (73.2%), were farmers, followed by retirees (17, 6.9%) and household workers and unemployed (17, 6.9%); 165 (69%) lived in hilly or mountainous areas and 62 (25.9%) in the plains; and the median age was 64 years (range 2-93 years).

Of these subjects, 28 (11.4%) were initially diagnosed as SFTS-suspected cases and were then excluded. Records of 24 cases showed that the reason for exclusion was negative detection of SFTSV RNA. The other 218 (88.6%) cases were never diagnosed as SFTS cases.

### Laboratory detection

**Fig. 1:**
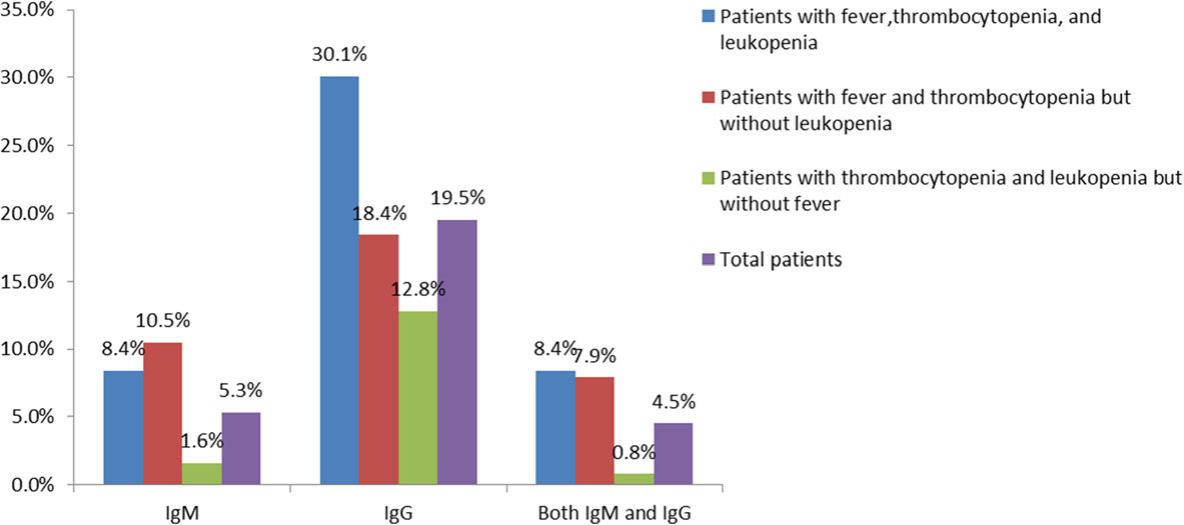
Seropositive rate of SFTSV antibodies in hospitalized patients with different clinical manifestations

Sera were collected from all 246 cases during the investigation. The median (interquartile range) days of specimen collection after disease onset was 346 d (250-484 d). In total, 50 (20.3%) cases were SFTSV antibody-positive, including 13 (5.3%) IgM-positive and 48 (19.5%) IgG-positive (the detailed SFTSV antibody detection results were showed in Additional file 1). Of the 13 IgM-positive cases, 11 (84.6%) were IgG-positive (9 with titres ≥1:400, 2 with titres equal to 1:100). Two specimens with IgG antibody against SFTSV (titres equal to 1:100) were identified in the serum samples of 150 patient-matched healthy control subjects from SFTS non-endemic areas, and no IgM was detected. The seropositive rates of antibodies were very high (8.4% for IgM and 30.1% for IgG) in patients with fever, thrombocytopenia and leukopenia (Fig. 1). Furthermore, among the IgG-positive cases in this group, 76% (19/25) of patients’ IgG antibody titres were greater than or equal to 1:400 (Table 1).

**Table 1:** SFTSV IgG antibody titres in hospitalized patients with different clinical manifestations

SFTSV-IgG titre (1:)	Patients with fever, thrombocytopenia, and leukopenia (*N* = 83) n (%)	Patients with fever and thrombocytopenia but without leukopenia (*N* = 38) n (%)	Patients with thrombocytopenia and leukopenia but without fever (*N* = 125) n (%)
6400	0(0)	0(0)	0(0)
1600	6(7.2)	2(5.3)	0(0)
400	13(15.7)	2(5.3)	4(3.2)
100	6(7.2)	3(7.9)	12(9.6)
Total	25(30.1)	7(18.5)	16(12.8)

Of the 28 patients who were excluded from the SFTS cases, 19 (67.9%) were IgG antibody seropositive, and the titres of 84.2% (16/19) IgG-positive cases were ≥1:400; 7 cases (25%) were both IgM and IgG antibody-positive. There was a significant difference between these 28 patients and healthy persons in the seropositive rate of IgG antibody (x^2^ = 100.347, *p* = 0.000). Of the 218 patients who had never been diagnosed with SFTS, 29 (13.3%) were IgG antibody-positive, and 37.9% (11/29) of the seropositive specimens had IgG titres ≥1:400; 6 cases (2.8%) were IgM antibody-positive, and 4 of them were both IgM and IgG antibody-positive. Table 2 showed the results of the SFTSV antibodies and titres in hospitalized patients with different clinical diagnoses.

**Table 2:** SFTSV antibodies in hospitalized patients with different clinical diagnoses

Diagnosis during hospitalization	No. detected	Seropositivity of IgM n(%)	Seropositivity of IgG n(%)	Titres of IgG (1:) n(%)				
				6400	1600	400	100	〈100
Patients who were not diagnosed with SFTS	218	6(2.8)	29(13.3)	0(0)	2(0.9)	9(4.1)	18(8.3)	189(86.7)
Patients who were initially diagnosed as SFTS-suspected cases and then excluded	28	7(25)	19(67.9)	0(0)	6(21.4)	10(35.7)	3(10.7)	9(32.1)
Total	246	13(5.3)	48(19.5)	0(0)	8(3.3)	19(7.7)	21(8.5)	198(80.5)

Sera samples collected during hospitalization were found for 14 hospitalized patients. The median age of these patients was 58 years old (range 27-81 years), and 7 (50%) were female. The median collection day after disease onset was 9 days. SFTSV RNA was not detected. The seropositive rates of SFTSV IgM, IgG, and the two antibodies simultaneously were 64.3% (9/14), 21.4% (3/14), and 14.3% (2/14), respectively. The 8 seropositive specimens of IgM had IgG antibody titres greater than or equal to 1:400 in the convalescent stage. The titres of IgG antibody against SFTSV were not higher than 100 in serum samples from the acute phase. Four-fold elevation of SFTSV IgG antibody titres or seroconversion was observed in 71.4% (10/14) of patients. Table 3 showed detailed information on these 14 cases.

**Table 3:** Clinical manifestations and SFTSV antibody detection results in the 14 hospitalized cases

Case No.	Gender	Lymphadenopathy	WBC	ALT	AST	CK	ALP	LDH	Days after onset^(AP)^	IgM^(AP)^	IgG^(AP)^	Days after onset^(CP)^	IgM^(CP)^	IgG^(CP)^
1	F	N	L	H	H	N	N	H	35	> 10	100	320	< 10	1600
2	F	N	N	H	H	H	N	H	3	< 10	100	290	< 10	100
3	F	N	L	N	N	–	N	H	5	< 10	< 100	282	< 10	< 100
4	F	N	L	H	N	N	N	N	5	< 10	< 100	313	< 10	< 100
5	M	N	L	H	H	H	N	H	1	> 10	< 100	386	< 10	400
6	M	N	N	H	H	H	H	H	13	> 10	< 100	407	> 10	1600
7	M	N	N	H	H	H	N	H	17	> 10	< 100	410	> 10	400
8	F	Y	L	N	H	N	N	N	4	< 10	< 100	373	< 10	100
9	M	N	L	H	H	H	L	H	9	> 10	< 100	423	< 10	400
10	F	Y	L	H	H	H	N	H	9	> 10	< 100	470	< 10	400
11	M	N	L	H	H	H	N	H	12	> 10	< 100	473	< 10	100
12	F	N	L	H	H	H	N	H	12	> 10	100	478	< 10	400
13	M	N	L	H	H	H	N	H	15	> 10	< 100	482	> 10	400
14	M	N	N	N	N	N	N	N	5	< 10	< 100	551	< 10	< 100

*F* female, *M* male, *N* no or normal, *Y* yes, *L* low, *H* high, *AP* acute phase of disease, *CP* convalescent phase of disease

### SFTS incidence estimation

The above results indicated that some patients’ diagnoses were missed. We speculated that the 27 patients (11%, 27/246) with IgG antibody titres greater than or equal to 1:400 in the retrospective study were most likely misdiagnosed. These patients did not significantly differ in major clinical signs (lymphadenopathy, WBC, AST, ALP, and LDH) from lab-confirmed SFTS cases (Table 4) [1]. Furthermore, 33.3% of 27 cases were also IgM-positive. Therefore, we estimated that the missed diagnosis rate was approximately 8.3% in high SFTS endemic areas (Table 5). Based on this finding, the actual SFTS incidence in high endemic areas is much higher than the current reporting number.

**Table 4:** Comparison of characteristics between the estimated missed diagnosis cases and lab-confirmed cases

Characteristic	Missed SFTS cases^c^ no. / total no. (%)	Lab-confirmed SFTS cases^b^ no. / total no. (%)	*p*-value
Age (yr)^a^	27/59(35-83)	/	/
Male	19/27(70.4)	/	/
Farmer	24/27(88.9)	/	/
Hills or mountains	25/27(92.6)	/	/
Fever	23/27(85.2)	100/100(100)	0.002
Lymphadenopathy	6/23(26.1)	23/81(28.4)	0.828
WBC decreasing	23/27(85.2)	64/74(86.5)	0.867
ALT elevation	18/27(66.7)	53/64(82.8)	0.040
AST elevation	24/27(88.9)	59/63(93.7)	0.440
ALP elevation	1/26(3.8)	3/53(5.7)	1.000
LDH elevation	22/26(84.6)	49/51(96.1)	0.171
CK elevation	18/23(78.3)	25/49(51)	0.028

^a^Presented as the median age and the range of age

^b^Characteristics of lab-confirmed SFTS cases were retrieved from a published article [1]

/:No data

^c^Patients with IgG titres**≥**1:400 were considered missed SFTS cases

**Table 5:** Estimated missed diagnosis rate of SFTS

Study sites	No. SFTS cases during investigation	No. missed SFTS cases^a^	Estimated missed diagnosis rate^b^
place1	195	19	8.9%
place2	104	8	7.1%
	299	27	8.3%

^a^Patients with IgG titres**≥**1:400 were considered missed SFTS cases

^b^Missed diagnosis rate = No. missed cases / (No. missed cases + No. SFTS cases during the investigation)×100%

## Discussions

SFTS was first reported in mainland China 7 years ago as a highly pathogenic disease. In 2010, China developed “guidelines for SFTS prevention and control (2010)” [15] to standardize the case diagnosis and report. Because of the limitations in early understanding of SFTS, missed diagnoses may exist. Therefore, we conducted this study to address these missed cases. Our results showed that an estimated 8.3% of SFTS diagnoses were missed in high endemic areas of China. According to this result, the actual incidence of SFTS is much higher than the currently reported level.

In this study, we chose two provinces as our study areas based on the following reasons. First, these two provinces were both high SFTS endemic areas. The total number of SFTS cases in these regions between 2011 and 2014 were 1074 and 458 [2], respectively. Second, these two provinces had very different SFTS types. The proportions of lab-confirmed cases, clinically diagnosed cases (although this classification was not officially defined), and suspected cases were 70.9%, 25.2%, and 3.9% in one province and 39.1%, 55.0%, 5.9% in the other, respectively [2]. Therefore, these differences might provide more information about SFTS diagnosis for this study. In this study, we just selected a site, locating in low incidence area in place1 and being adjacent to some of the study sites, to understand the most basic level of SFTSV infection. SFTS was mainly characterized by fever, thrombocytopenia and leukopenia, especially the former two [1, 23]. To make the study more targeted, all the subjects in our study had thrombocytopenia. Meanwhile, considering the presence of mild cases, we included patients without fever in this investigation.

The SFTSV IgG seropositive rate (20.5%) in all subjects, especially in patients with fever, thrombocytopenia and leukopenia (30.1%), was higher than the rate reported in high endemic areas in healthy people; for example, these rates were 7.2% in Zhejiang province [14], 6.6% in Xinyang, Henan province [24], 5% in Macheng, Hubei province [25], 4.7% in the western region of Anhui province [26], 3.3% in Laizhou, Shandong province [27], and 0.4% in seven counties of Jiangsu province [28]. We found that the IgM positive rate was also high, which was not observed in healthy people [25]. Additionally, 69.2% of SFTSV IgM-positive cases had IgG titres ≥1:400.

A high seroprevalence (67.9% for IgG, 25% for IgM) was observed in the 28 hospitalized patients (initially diagnosed as suspected SFTS cases and then excluded). Of these patients, 57.1% had IgG antibody titres greater than or equal to 1:400. In addition, 64.3% of the 14 sera collected in the acute phase were SFTSV IgM-positive, and this rate was similar to that described in lab-confirmed SFTS cases [29, 30]. This phenomenon suggested that some diagnoses of SFTS cases might be missed because of an over-reliance on laboratory test results [2]. SFTSV RNA test by real time RT-PCR was the most commonly used method for confirmation of recent SFTSV infection [1, 29]. However, many factors such as a long specimen collection time after disease onset [30–32], poor specimen and preservation condition, unskilled operation, and low virus load can lead to a low detection of viral RNA. Furthermore, of the 218 hospitalized patients who were never diagnosed with SFTS, 13.3% were IgG-positive and 2.8% IgM-positive; 13.7% of IgG-positive specimens were positive for IgM. A small portion cases had IgG titres ≥1:400. We speculated that the diagnoses of these patients may have been missed because of their mild or subclinical manifestations [14]. So we suggested the use of serological detection methods during acute phase also, in order to increase the diagnostic sensibility for SFTS.

In our study, 27 of 246 hospitalized patients in our study were considered to be missed cases in light of their high IgG titres, high IgM-positive rate and the similarity of their clinical characteristics with lab-confirmed SFTS cases. An estimated 8.3% of SFTS diagnoses in study sites were missed. However, our study was a preliminary study on missed diagnosis and the estimation was conservative. Further studies should be conducted to understand the reasons for these missed cases to implement measures (such as adding a new SFTS case category - “SFTS clinical diagnosis cases” or using serological detection methods during acute phase) to prevent missed diagnoses.

Current studies have shown that SFTSV IgM and IgG antibodies can still be detected 1.3 years and over 3 years after SFTSV infection [33, 34], respectively. In addition, detection of sera antibodies can be an alternative or supplement for later-collected specimen due to their later appearance than viremia [31]. However, several limitations of this study should be clarified. First, most of the specimens collected during the retrospective investigation were 6 months to 1.5 years after disease onset. SFTSV antibodies in some specimens may disappear or the titres may decrease [33], which can affect the antibody-positive rate. Second, we focused on high SFTS endemic provinces only; other non-endemic or low endemic provinces were not involved because of the time constraints. Accordingly, the analysis of missed SFTS diagnosis in China was not comprehensive. In further studies, the analysis should include other areas.

## Conclusions

In conclusion, by analysing the clinical manifestations, epidemiological data, and SFTSV infection of hospitalized patients, our study preliminarily estimated a rate of missed SFTS diagnosis. The results showed that the SFTS incidence was much higher than the currently reported level. Reclassification of SFTS case types and improvements in detection capacity should be highlighted to ensure better SFTS prevention and control.

## Additional file

Additional file 1: SFTSV antibody detection results of 246 patients. The detailed IgM and IgG antibody OD value of sera collected from 246 patients were showed in this Additional file 1. (XLSX 26 kb)

## Abbreviations

ALT: Alanine aminotransferase
AP: Acute phase of disease
AST: Aspartate transaminase
CK: Creatine kinase
CP: Convalescent phase of disease
HRP: Horseradish peroxidase
LDH: Lactate dehydrogenase
OD: Optical density
PLT: Platelet
RT-PCR: Reverse transcription-polymerase chain reaction
SFTS: Severe fever with thrombocytopenia syndrome
SFTSV: SFTS virus
WBC: White blood cell
